# Systematic review of guideline-recommended medications prescribed for treatment of low back pain

**DOI:** 10.1186/s12998-022-00435-3

**Published:** 2022-05-13

**Authors:** Morgan R. Price, Zachary A. Cupler, Cheryl Hawk, Edward M. Bednarz, Sheryl A. Walters, Clinton J. Daniels

**Affiliations:** 1 VA Puget Sound Health Care System, Seattle, WA USA; 2 Butler VA Health Care System, Butler, PA USA; 3 Texas Chiropractic College, Pasadena, TX USA; 4 VA Puget Sound Health Care System, Tacoma, WA USA; 5 Logan University, Chesterfield, MO USA

**Keywords:** Systematic review, Clinical practice guideline, Low back pain, Drug therapy

## Abstract

**Objective:**

To identify and descriptively compare medication recommendations among low back pain (LBP) clinical practice guidelines (CPG).

**Methods:**

We searched PubMed, Cochrane Database of Systematic Review, Index to Chiropractic Literature, AMED, CINAHL, and PEDro to identify CPGs that described the management of mechanical LBP in the prior five years. Two investigators independently screened titles and abstracts and potentially relevant full text were considered for eligibility. Four investigators independently applied the Appraisal of Guidelines for Research and Evaluation (AGREE) II instrument for critical appraisal. Data were extracted for pharmaceutical intervention, the strength of recommendation, and appropriateness for the duration of LBP.

**Results:**

316 citations were identified, 50 full-text articles were assessed, and nine guidelines with global representation met the eligibility criteria. These CPGs addressed pharmacological treatments with or without non-pharmacological treatments. All CPGS focused on the management of acute, chronic, or unspecified duration of LBP. The mean overall AGREE II score was 89.3% (SD 3.5%). The lowest domain mean score was for *applicability,* 80.4% (SD 5.2%), and the highest was *Scope and Purpose,* 94.0% (SD 2.4%). There were ten classifications of medications described in the included CPGs: acetaminophen, antibiotics, anticonvulsants, antidepressants, benzodiazepines, non-steroidal anti-inflammatory drugs (NSAIDs), opioids, oral corticosteroids, skeletal muscle relaxants (SMRs), and atypical opioids.

**Conclusions:**

Nine CPGs, included ten medication classes for the management of LBP. NSAIDs were the most frequently recommended medication for the treatment of both acute and chronic LBP as a first line pharmacological therapy. Acetaminophen and SMRs were inconsistently recommended for acute LBP. Meanwhile, with less consensus among CPGs, acetaminophen and antidepressants were proposed as second-choice therapies for chronic LBP. There was significant heterogeneity of recommendations within many medication classes, although oral corticosteroids, benzodiazepines, anticonvulsants, and antibiotics were not recommended by any CPGs for acute or chronic LBP.

**Supplementary Information:**

The online version contains supplementary material available at 10.1186/s12998-022-00435-3.

## Introduction

Low back pain (LBP) is the single largest cause of years lived with disability in the world [1]. Sixty-eight percent of physician visits in the United States (US) involve drug therapy and the most frequently prescribed therapeutic classes include analgesics, antihyperlipidemic agents, and antidepressants [2]. Each year there are 860.5 million drugs prescribed in the US and the world’s population receives 4 trillion doses of medication [2–4]. Between 2015 and 2018, in 30 day windows, nearly half of adults in the US used at least one prescription. A quarter of adults in the US and United Kingdom (UK) reported taking 3 or more medications, and more than one-tenth of US adults were taking five or more medications [2, 5].

Low back pain is often managed by primary care providers with medication recommendations to include non-steroidal anti-inflammatory drugs (NSAIDs), acetaminophen, and opioids [6–8]. Primary care providers, in concordance with clinical practice guidelines (CPG), also encourage non-pharmacological LBP care to include patient education, remaining physical active, and manual therapies. Integration of non-prescribing providers (e.g. chiropractors) who manage LBP into health care facilities has demonstrated high satisfaction and the potential to play a role in reducing opioid use for pain [9–12]. Although in most instances, these providers do not directly manage medications, they will commonly encounter patients who are managed concurrently with pharmaceuticals for their LBP. While managing LBP, these non-prescribing providers may encounter relevant questions from patients, or the medications may have implications on their clinical decision making. Many chiropractors in non-prescribing jurisdictions report advising or recommending patients take analgesics, non-steroidal anti-inflammatory drugs (NSAIDs), or other over-the-counter medications [13–15]. This underscores the need for non-prescribing providers managing LBP to be familiar with various national- and association-developed clinical practice guideline (CPG) medication recommendations.

As the scientific literature is continually evolving, appreciating and understanding the most current CPGs for the pharmaceutical management of LBP is challenging. This may be particularly true to non-prescribing providers, and therefore, they may be less inclined to follow the relevant literature. The purposes of this study were to (1) systematically evaluate the literature for CPGs that included the pharmaceutical management of non-specific LBP; (2) appraise the methodological quality of the CPGs; (3) qualitatively synthesize the recommendations with the intent to inform non-prescribing providers who manage LBP.

## Methods

The review was conducted in accordance with the Preferred Reporting Items for Systematic Reviews and Meta-Analysis (PRISMA) [16].

### Eligibility criteria

The inclusion and exclusion criteria are outlined in Table 1. All CPGs published by peer-reviewed journals in English were included in the search. All other study designs were excluded. We restricted our search of guidelines to articles from the past five years (11/2015—11/2020), as it has been recommended that CPGs be updated every 3 to 5 years [17–19], and we desired to include as many recent CPGs as possible.

**Table 1:** Eligibility Criteria

Inclusion	Exclusion
Published 11/06/2015–11/06/2020 English language Guidelines related to low back pain Provides recommendations on oral medication	Non-English language Nonrelevant Not a guidelines (e.g. systematic reviews) Not related to non-cancer, musculoskeletal low back pain Specific to structural origins of lower back pain (e.g. lumbar spinal stenosis, lumbar disc herniation) Does not include recommendations for oral medication Published before November 2015 Failed back surgery syndrome Inflammatory spondyloarthropathies (e.g. rheumatoid arthritis, ankylosing spondylitis, psoriatic arthritis) Guidelines specific to injectable medications

### Information sources

A literature search was performed of PubMed, Cochrane Database of Systematic Reviews, Index to Chiropractic Literature, Allied and Complementary Medicine Database (AMED), Cumulative Index for Nursing and Allied Health Literature (CINAHL), and Physiotherapy Evidence Database (PEDro). The search was conducted in November 2020.

### Search strategy

We combined numerous search terms relevant to clinical guideline recommendations for pharmaceutical management of LBP (Table 2). The list of references of included publications were manually searched for additional guidelines potentially meeting the inclusion criteria.

**Table 2:** Search terms

Condition/region	Treatment Strategy	Filter
Back	Pharmaceutical	Guidelines
Lumbar	Medication	Best practices
Lumbopelvic	Pharmacology	Care pathway
Sciatic pain		Clinical recommendations

### Selection process

Title and abstract screening was independently conducted by two authors (M.R.P and E.M.B.) for each article and subsequently saved if eligibility criteria was possibly met. Full papers were retrieved and reviewed independently by two authors (M.R.P and Z.A.C.) to verify inclusion criteria. A third author (C.J.D) was consulted in the case of disagreements.

### Data collection process

Two authors (M.R.P and C.J.D) independently performed data extraction for the included studies. When consensus was not reached a third author (Z.A.C.) adjudicated.

### Data items

Items collected on the data extraction tables included: stage of LBP recommendations (i.e. acute, subacute, chronic), medication classification, recommendation for use (i.e. recommended for, recommended against, inconclusive), dosages and utilization, contraindications, harms, quality of evidence, and strength of recommendation. If a CPG did not report on a pre-determined category for extraction, the item was notated as unavailable.

### Study risk of bias assessment

Risk of bias was assessed by four authors (M.R.P., C.J.D., Z.A.C., and C.H.) utilizing the Appraisal of Guidelines for Research and Evaluation (AGREE) II tool [20]. The AGREE II is a widely utilized assessment integrating 23 items divided across 6 domains (Table 3) followed by an overall assessment quality [21]. Using the AGREE II criteria, the individual items within each domain of each article were independently scored on a scale of 1–7 (1 equals strongly disagree to 7 equals strongly agree). Reviewers underwent standardized online training provided on the AGREE website prior to scoring [22].

**Table 3:** The AGREE II Instrument

AGREE II domains and items	
*Domain 1. Scope and Purpose*	
1. The overall objective(s) of the guideline is (are) specifically described	
2. The health question(s) covered by the guideline is (are) specifically described	
3. The population (patients, public, etc.) to whom the guideline is meant to apply is specifically described	
*Domain 2. Stakeholder Involvement*	
4. The guideline development group includes individuals from all relevant professional groups	
5. The views and preferences of the target population (patients, public, etc.) have been sought	
6. The target users of the guideline are clearly defined	
*Domain 3. Rigor of Development*	
7. Systematic methods were used to search for evidence	
8. The criteria for selecting the evidence are clearly described	
9. The strengths and limitations of the body of evidence are clearly described	
10. The methods for formulating the recommendations are clearly described	
11. The health benefits, side effects, and risks have been considered in formulating the recommendations	
12. There is an explicit link between the recommendations and the supporting evidence	
13. The guideline has been externally reviewed by experts prior to its publication	
14. A procedure for updating the guideline is provided	
*Domain 4. Clarity of Presentation*	
15. The recommendations are specific and unambiguous	
16. The different options for management of the condition or health issue are clearly presented	
17. Key recommendations are easily identifiable	
*Domain 5. Applicability*	
18. The guideline describes facilitators and barriers to its application	
19. The guideline provides advice and/or tools on how the recommendations can be put into practice	
20. The potential resource implications of applying the recommendations have been considered	
21. The guideline presents monitoring and/or auditing criteria	
*Domain 6. Ethical Independence*	
22. The views of the funding body have not influenced the content of the guideline	
23. Competing interests of guideline development group members have been recorded and addressed	

AGREE II, Appraisals of Guidelines for Research and Evaluation, Version II

### Synthesis methods

The scores for each item were entered into a Microsoft Excel 365 (Redmond, WA) spreadsheet, with scores for each domain represented as the cumulative score of all items divided by the maximum possible domain score per reviewer [23]. All reviewers scores were summated per domain with percentage calculated based on the maximum score possible for each respective domain.

We synthesized recommendations using evidence tables (Additional file 1: Appendix S1 and Additional file 2: Appendix S2). AGREE II does not provide cut-offs to differentiate CGP study quality (e.g. high, acceptable, low quality), therefore we followed prior studies that deemed any guidelines with domain scores less than 50% as low quality [23–25]. Recommendations were categorized by medication class and summarized according to whether an intervention was (1) recommended, (2) not recommended, (3) inconclusive due insufficient or conflicting evidence, or (4) not assessed or addressed by the CPG, and stratified by duration of LBP (i.e., acute/subacute, or chronic) (Tables 4 and 5). We considered a medication to be “recommended” if the CPG included terminology such as: “strongly recommended”, “recommended for”, “strong for”, “suggested for”, “first-line treatment”, “second-line treatment”, or “recommend for consideration”. We considered a medication to be “not recommended” if the CPG included terminology such as: “recommend against”, “strongly against”, “weak against”, or “do not routinely offer”. We considered medication recommendations inconclusive if they used terminology such as: “inconclusive”, “no recommendation”, or insufficient evidence”. Tramadol is deemed a “synthetic opioid” or “atypical opioid” and, as a schedule IV drug in the United States, has been thought to have less potential for abuse and dependence compared to other opioids [26]. Buprenorphine and tapentadol are also included in this medication class [27, 28].

**Table 4:**
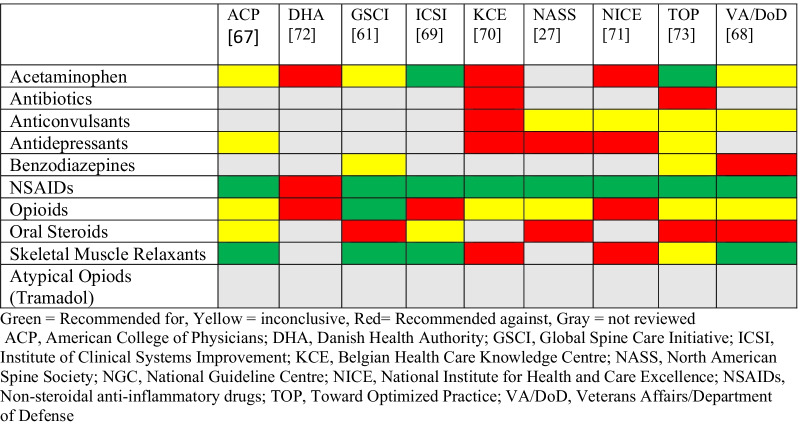
Evidence table—acute low back pain recommendations by guideline

**Table 5:**
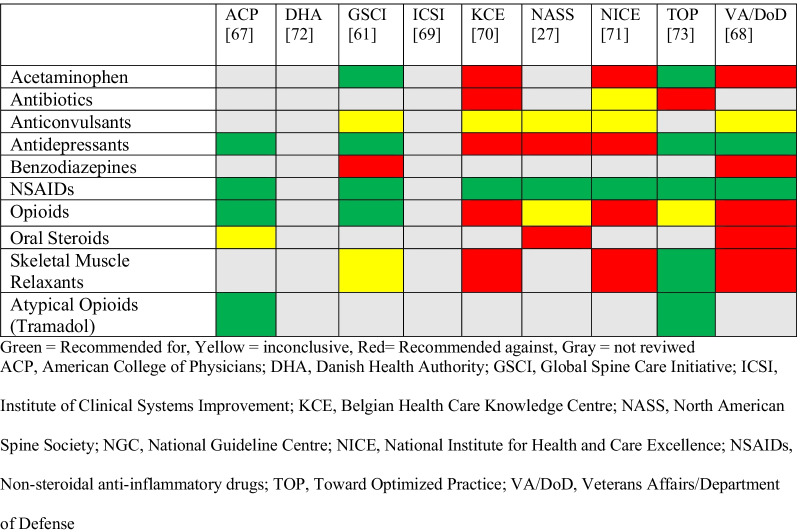
Evidence table—chronic low back pain recommendations by guideline

## Results

### Study selection

**Fig. 1:**
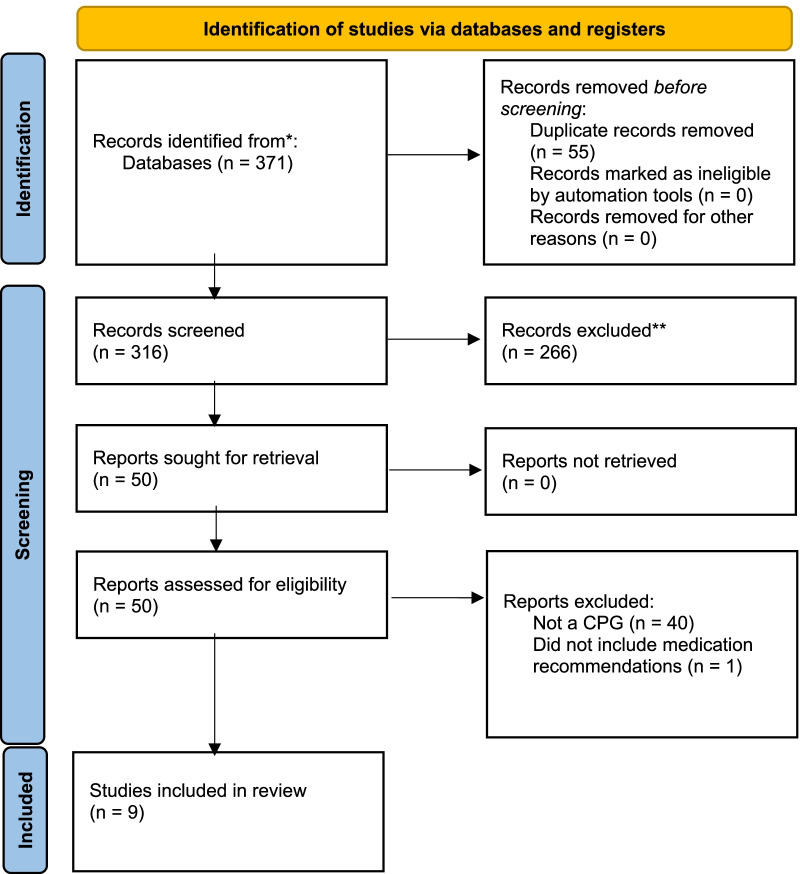
PRISMA 2020 flow diagram for new systematic reviews which included searches of databases and registers only

A comprehensive database search revealed 316 distinct records, from which 50 full-text were subsequently retrieved and reviewed (Fig. 1). Nine citations met the eligibility criteria for inclusions. Reasons for full-text exclusion included “not a CPG” [23, 29–66] and “does not include medication recommendations” [67].

### Risk of bias in studies

Quality was assessed across six domains and an overall assessment: scope and purpose (range 80%-100%), stakeholder involvement (range 48–100%), rigor of development (range 53–100%), clarity of presentation (range 89–100%), applicability (range 52–99%), editorial independence (range 29–100%), and overall assessment (range 71–100%) (Table 6). The mean overall AGREE II score across CPGs was 89.3% (SD 3.5%). The lowest mean score was for *applicability,* 80.4% (SD 5.2%), and the highest was *Scope and Purpose,* 94.0% (SD 2.4%).Of the included CPGs, overall assessment of eight were rated as high-quality [62, 68–74], and one was rated as low-quality [75].

**Table 6:** CPG AGREE II domain scores and quality assessments reported as percentage

Title	1. Scope and Purpose	2. Stakeholder Involvement	3. Rigour of development	4. Clarity of Presentation	5. Applicability	6. Editorial Independence	Overall Assessment	Quality (High/Low)
ACP [66]	95	92	98	95	88	100	96	High
DHA [72]	94	88	99	92	52	98	86	High
GSCI [60]	94	86	77	96	88	100	89	High
ICSI [69]	96	98	86	99	87	95	86	High
KCE [70]	99	100	99	98	93	100	96	High
NASS [68]	100	100	95	89	71	100	96	High
NICE [71]	100	99	99	98	99	100	100	High
TOP [73]	80	48	53	100	64	29	71	Low
VA/DoD [67]	95	95	88	98	82	100	96	High

ACP, American College of Physicians; DHA, Danish Health Authority; GSCI, Global Spine Care Initiative; ICSI, Institute of Clinical Systems Improvement; KCE, Belgian Health Care Knowledge Centre; NASS, North American Spine Society; NGC, National Guideline Centre; NICE, National Institute for Health and Care Excellence; TOP, Toward Optimized Practice; VA/DoD, Veterans Affairs/Department of Defense

### Results of syntheses

#### Description of the guidelines

Ten classifications of medications were described including: acetaminophen, antibiotics, anticonvulsants, antidepressants, benzodiazepines, non-steroidal anti-inflammatory drugs (NSAIDs), opioids, oral corticosteroids, skeletal muscle relaxants (SMRs), and atypical opioids. No CPGs provided explicit recommendations regarding subacute LBP. Rather, when addressed, the CPGs treated subacute similar to acute and organized as a single recommendation.

#### Non-steroidal anti-inflammatory drugs

Nine guidelines addressed NSAIDs for LBP [68, 62, 69–75] and NSAIDs were the class of medications most commonly recommended (8/9 CPGs). Three guidelines specifically described ibuprofen [73–75], two described diclofenac [73, 75], one described naproxen [74], and one listed piroxicam, etoricoxib, and indomethacin [73]. Six CPGs [68, 62, 69–72] did not recommend any specific NSAIDs, and one stated there was insufficient evidence to recommend the use of NSAIDs [68].

The strength of recommendations ranged from weak to strong with very low to moderate quality evidence cited. NSAIDs were recommended as the first pharmacological choice for acute LBP by one CPG [69] and the first choice for chronic LBP by two CPGs [62, 69]. Following acetaminophen, NSAIDs were recommended as a second-choice medication for both acute and chronic LBP in one CPG [75].

Five CPGs recommended NSAIDs for acute LBP [62, 69–71, 74, 75] while four CPGs recommended them for chronic LBP [62, 69, 70, 75], and three CPGs recommended them for unspecified durations of LBP [68, 72, 73]. Specific dosage was discussed in two CPGs. Ibuprofen dosage ranged from 1,200–1,800 mg per day [74] to 2,400 mg per day with a maximum of 3,200 mg per day [75]. Two CPGs [69, 72] recommended that NSAIDs should be taken at the lowest effective dose for the shortest period of time to minimize the potential for harm. Three other CPGs additionally supported only short-term use [71, 73, 75].

One CPG [74] had a weak recommendation against NSAIDs for acute LBP, stating that the evidence points toward no short-term effect. Seven CPGs discussed potential risks or side effects which included gastrointestinal, cardiovascular, renal, and/or hepatic concerns [62, 62, 69, 70, 72, 73, 75], and two CPGs [73, 75] stated that adults over 45 years of age should utilize gastric protection with a proton-pump inhibitors when taking NSAIDs.

#### Skeletal muscle relaxants

Skeletal muscle relaxants (SMRs) were reviewed by seven CPGs [62, 69–73, 75]. Cyclobenzaprine [70, 75] and tizanidine [72, 73] were the most discussed SMRs. Only one CPG discussed specific SMR dosage, indicating that 10-30 mg/day of cyclobenzaprine demonstrated the greatest benefit within one week in the presence of acute LBP or flare-up of chronic LBP with prominent muscle spasm [75].

Five CPGs recommended SMRs for acute LBP (5/7 CPGs); of these, one recommended them as a first-line pharmacological therapy [69] and two stated only to consider once NSAIDs and acetaminophen had failed [62, 75]. Two CPGs recommended against the use of SMRs for all LBP [72, 73], one CPG recommended against them for chronic LBP in the absence of episodic exacerbations [75], and one CPG reported inconclusive evidence to make a recommendation for chronic LBP [62]. Five guidelines agreed that if SMRs are going to be utilized, it should only be for a short-term duration (less than 2 weeks) [62, 70–72, 75]. Two of these CPGs specifically recommended less than 1 week duration of SMRs [71, 75] and one CPG went on to state there was no evidence for the long-term use of SMRs [70].

The strength of recommendations among CPGs that included SMRs ranged from strong against for all durations of LBP [72] to strong for as a first line pharmacological choice in the treatment of acute LBP [69]. Two CPGs noted the conflicting evidence in the literature for the use of SMRs in acute LBP and reported an increased incidence of adverse events in patients treated with SMRs compared to placebo [72, 73].

Potential harms described included sedation, drowsiness, potential for abuse, dry mouth, driving impairment, nausea, dizziness, and headache [69–71, 73, 75]. One CPG commented that patients may be anxious as a result of sudden disabling back pain and could have difficulty relaxing with the result of muscle spasm, however, this did not justify the use of SMRs [72].

#### Opioids

All nine CPGs discussed opioids for LBP [68, 62, 69–75]. Five CPGs considered opioids as a potential treatment for acute LBP (5/9 CPGs), but indicated they should be prescribed with caution, only utilized for short-term duration, and in the presence of serious pathology and severe pain which has failed other pharmacological methods [68, 62, 69, 72, 75]. One CPG discussed trialing weak opioids (i.e. codeine) before considering stronger opioids such as morphine sulfate, hydromorphone HCl, oxycodone HCl, or a fentanyl patch [75]. One guideline highlighted using long-acting opioid choices for chronic LBP as preferred to short-acting [75], and others recommended initial trials of opioids not exceed 3 days [71] or range from 1–14 days [74].

Two CPGs agreed that there is insufficient evidence regarding acute LBP to recommend use of opioids [69, 70]. Three CPGs recommended against the utilization of all opioids stating that the risk of opioids outweigh the relief that they may provide [71, 73, 74]. Two CPGs agreed that the evidence was lacking for long-term use of opioids for chronic LBP [70, 75]. Potential side effects of opioids included the imminent concerns of death, physical dependence, addiction, nausea, dizziness, headache, nausea, somnolence, constipation, dry mouth, dizziness, opiate-induced hyperalgesia, endocrinological changes, and tolerance [62, 71, 72, 75].

#### Atypical opioids

Many CPGs discussed tramadol within the context of opioid medication, however, tramadol is widely considered an atypical opioid due to having opioidergic, noradrenergic, and serotonergic properties [28]. Three CPGs specifically separated tramadol out from opioids in their recommendations [68, 69, 75]. Two CPGs recommended utilization of tramadol for chronic LBP (2/3 CPGs), one a second choice [69] and another as a fourth choice option [75]. No CPGs specified strength of recommendations for tramadol. Caution was advised when prescribing tramadol in addition to a tricyclic antidepressant (TCA) or serotonin and norepinephrine reuptake inhibitor (SNRI) with a suggestion to slowly titrate to a maximum of 400 mg per day [75]. Potential side effects listed were dizziness, drowsiness, asthenia, gastrointestinal complaints, and risk for potential hypoglycemia [75].

#### Acetaminophen

Eight CPGs reviewed acetaminophen, also known as paracetamol, for LBP [62, 69–75]. Three guidelines recommended acetaminophen as an option for LBP (3/8 CPGs), with one recommending as a first choice for acute and chronic LBP [75]; one recommended as a first line treatment for chronic LBP but inconclusive for acute LBP [62]; and one recommended as an option for acute and subacute LBP with concurrent counseling on side effects [71].

Four CPGs recommended against the use of acetaminophen for LBP. One CPG did not specify status acute versus chronic LBP and recommended against routinely offering for LBP with or without leg pain [72]; one indicated there was no evidence to support acetaminophen for acute LBP and recommended not to use for long-term care [73]; one assessed for acute and subacute LBP and provided a weak recommendation against while indicating that acetaminophen should only be offered for new onset LBP in addition to usual care, as evidence points towards no short-term effect [74]; and one recommended against long-term use and cited insufficient evidence [76, 77] to recommend for or against time limited (less than 7 days) usage [70].

There was one CPG that was inconclusive for acute LBP to provide a recommendation [69]. They cited low-quality evidence that acetaminophen was not superior to placebo for pain intensity or function at 4 weeks [76], but concluded that there was insufficient evidence to make a recommendation determination [69].

Strength of recommendations were rated by three CPGs as weak against for acute LBP [69, 72, 74] and strong against for chronic LBP by one CPG [70]. Acetaminophen may be clinically indicated for pain relief [71] when NSAIDs [62, 72] and opioids [72] are not appropriate (i.e. kidney failure or history of substance use disorder). One CPG recommended 3000 mg per day as the maximum [75] and another suggested taking between 2000 and 4000 mg per day [74]. Potential contraindications and harms for acetaminophen included liver disease with long-term use [70, 71, 75], but considered by some to be small or negligible [62, 75].

#### Anticonvulsants

Six CPGs evaluated anticonvulsants, also known as antiepileptics [68, 62, 70, 72, 73, 75]. Four CPGs addressed specific anticonvulsants: gabapentin [62, 70, 73, 75], pregabalin [62, 70], and topiramate [73, 75]. None of the CPGs recommended anticonvulsants for acute or chronic LBP (0/6 CPGs). Two CPGs stated that there was insufficient evidence for or against anticonvulsants with acute LBP, with or without leg pain [70, 75]; two stated similar for chronic, as well [62, 70]. Three CPGs did not differentiate between acute and chronic LBP, but, of those, two reported insufficient evidence to make a recommendation [68, 73], and one recommended against prescribing anticonvulsants for LBP of any duration, with or without radicular pain in the absence of a neuropathic pain component [72].

Strength of recommendation was rated as strong against anticonvulsant use for all LBP by one CPG [72]. Gabapentin and pregabalin may provide small, short-term benefits, but due to the significant side effect possibilities, they did not make a recommendation [78, 79]. Specific dosages were not discussed. Common side effects reported were fatigue, dry mouth loss of balance, difficulty with mental concentration, memory and visual accommodation, and potential for abuse and dependence[70].

### Antidepressants

Antidepressants for LBP were evaluated by seven CPGs [68, 62, 69, 70, 72, 73, 75]. Six of the CPGs differentiated recommendations based on different classes of antidepressants or listed specific medications (i.e. TCAs, SNRIs, selective serotonin reuptake inhibitors (SSRIs)); the remaining CPG provided one blanket recommendation [68]. The SNRI, duloxetine, was the most frequently discussed antidepressant [69, 70, 75] followed by the TCA, amitriptyline [72, 75].

Regarding acute LBP, two CPGs reported insufficient evidence to make a recommendation [69, 75] and one recommended against the use of antidepressants [72]. For chronic LBP, three CPGs gave a weak recommendation in favor of duloxetine as a second-line pharmacological choice (3/7 CPGs) [62, 69, 70], and one stated that there was insufficient evidence for duloxetine, but instead recommended TCAs as a third-line pharmacological choice for chronic LBP [75]. One CPG recommended consideration of either SNRIs or TCAs when concomitant depression or anxiety was present with chronic LBP [62].

Three other CPGs recommended against the routine use of prescribing antidepressants for any LBP [68, 72, 73]. One CPG differentiated guidance based on medication subclass with a strong recommendation against the use of SSRIs and a weak recommendation against the use of TCAs or SNRIs [72]. The same CPG acknowledged, that TCAs and SNRIs demonstrated some clinical benefit, specifically the SNRI, duloxetine, for patients with chronic LBP and neuropathic pain. Specific dosage was not discussed. There was concern of potential for clinical harms from antidepressants [72] with contraindications of pre-existing cardiac abnormalities and glaucoma, and side effects such as drowsiness and anticholinergic effects [75].

### Benzodiazepines

Four CPGs reviewed benzodiazepines [62, 70, 71, 75] and none recommended as a treatment for LBP. Two CPGs recommended against benzodiazepines for chronic LBP [62, 70]; one recommended against use for acute LBP [70]. Two CPGs were inconclusive for utilization in acute LBP [62, 75] and one did not give specific recommendations though stated that they should rarely be used, citing potential harms associated, and if so, for less than one week duration [71]. Specific benzodiazepine medications and dosage was not discussed.

Strength of recommendations were rated as strong against by one CPG [70]. Benzodiazepines are contraindicated for geriatric patients due to the sedative hypnotic side effects [71] and there is potential for serious side effects including sedation, potential for abuse, overdose, death due to respiratory depression, somnolence, fatigue, and lightheadedness [62, 70, 71]. One CPG listed serious pathology (e.g. cancer, infection, cauda equina syndrome) as the only indication for prescribing benzodiazepines for acute LBP [62] and another CPG proposed a short course of benzodiazepines if acetaminophen or NSAIDs have failed to improve acute LBP [75].

### Oral corticosteroids

Six CPGs considered oral corticosteroids and none recommended for the treatment of LBP [68, 62, 69–71, 75]. Two CPGs recommended against the use of oral corticosteroids for acute LBP [62, 75] and one CPG recommended against their use for chronic LBP [70]. One CPG additionally recommended against their use for any duration of LBP [68]; another did not give any specific recommendations, but did state that there was low-quality evidence in light of oral corticosteroids providing no effect on both pain or function [71]. Finally, one CPG [69] stated that there was insufficient evidence to make recommendations regarding both acute and chronic LBP, although evidence showed no difference compared with placebo in acute [80, 81]. Although no CPGs recommended oral corticosteroids for axial low LBP, a commonly cited randomized controlled trial (RCT) [81] in the CGPs did demonstrate modest improvement in function in patients with acute radiculopathy secondary to disc herniation, but did not demonstrate improvement in pain or incidence of surgery.

Strength of recommendations were rated as strong against by one CPG [70]. Due to the overwhelming recommendation against use, there was no dosage or titration guidance provided. Several of the CPGs discussed potential risks or side effects including: osteonecrosis, mood changes, anxiety, blurred vision, numbness or tingling in the arms or legs, swelling of extremities, insomnia, appetite changes, increased sweating, acne, nervousness, joint pain, headache, and indigestion [62, 69–71].

### Antibiotics

Three CPGs reviewed oral antibiotics, and none recommended for LBP [72, 73, 75]. Two CPGs recommended against the use of antibiotics for unspecified durations of LBP [72, 75] and one CPG stated it could not make a recommendation due to insufficient evidence [73]. The strength of recommendations cited were moderate to very low to expert opinion. None of the CPGs provided recommendations in favor of antibiotics and thus there was no guidance related to dosage or titration. The single RCT cited demonstrated an improvement in health utilization but also an increase in adverse events in chronic LBP with MRI confirmed disc prolapse [82].

## Discussion

### Main findings

Nine CPGs investigated 10 unique therapeutic medication classes for the management of LBP. NSAIDs were the most frequently endorsed medication for the treatment of both acute and chronic LBP as a first line pharmacological therapy. Acetaminophen and SMRs were inconsistently recommended for acute LBP. Meanwhile, with less consensus among CPGs, acetaminophen and antidepressants were proposed as second-choice therapies for chronic LBP. There was significant heterogeneity of recommendations between many medication classes, although oral corticosteroids, benzodiazepines, anticonvulsants, and antibiotics were not recommended by any CPGs for acute or chronic LBP.

### Comparison with the literature

Nearly all guidelines the included CPGs recommended non-pharmacological treatments for non-specific LBP, however it was not always delineated as to precede or be used in conjunction with pharmacological intervention [62, 69–75]. Although not the objective of this systematic review, recommendation for non-pharmacologic treatment is worth highlighting as early exposure to non-guideline concordant care (e.g. opioids) may predispose patients in transitioning from acute to chronic low back [8].

A potential contributor to the disagreement between CPG recommendations could be the lack of high-quality evidence to inform the literature, as many CPGs cited relatively few RCTs. Therefore, there is increased weight on expert clinical opinion to interpret lower quality evidence and generate recommendations. Other factors involved in formulating recommendations involve balancing benefit versus harm, and considerations for cost-effectiveness and feasibility. This enhances the possibility of interpretation bias or varying importance placed on these different considerations. The subsequent recommendations may be more indicative of the potential for lack of harm and ease of accessibility rather than evidence of effectiveness derived from prospective RCTs.

For example, a RCT of more than 1,100 patients found acetaminophen to be not better than placebo for recovery time, pain, disability, function, global system change, sleep, or quality life [76]. This study was cited by six of the included CPGs, with three recommending acetaminophen; one of which recommending as a first-line choice for acute LBP and two recommending first-line for chronic LBP. A Cochrane systematic review [77] evaluated only two RCTs [76, 83] and determined acetaminophen to be ineffective for the management of acute LBP. The Cochrane review stated, “the high-quality evidence and precise estimate of no effect for acute LBP suggest that no additional trials of paracetamol (acetaminophen) for acute LBP are required.” Additionally, the Cochrane group noted there were no trials evaluating acetaminophen use in chronic LBP and thus it was impossible to make a recommendation.

For acetaminophen, a lack of high-quality clinical evidence and variability in recommendations from the same pool of evidence requires evaluating the methods and included citations amongst the CPGs. Two of the three CPGs that recommended acetaminophen [71, 75] listed their supporting citations as reviews [84, 85] rather than RCTs used for evidence in the other five CPGs [69, 70, 72–74]. The remaining CPG that recommended acetaminophen was specific to low- and middle-income communities, and was influenced by the low cost and small harms associated while conceding “the role of acetaminophen for temporary relief of acute spine pain is uncertain.” [62] Potential harm to benefit ratio weighs heavily in CPG recommendation generation and may influence variability in recommendations with limited high-quality clinical evidence [86]. This harm versus benefit decision making was highlighted in a recent systematic review [46] where associated harms were described to greatly outweigh the potential for benefit in patients with non-specific subacute and chronic LBP, despite some clinical benefit identified with acute LBP [84]. It is difficult to interpret the recommendations for tramadol, however, as it is commonly grouped with all opioid medications. Only two CPGs independently discussed tramadol for chronic LBP [69, 75]. Both CPGs cited two RCTs [87, 88] in supporting a recommendation against tramadol as a first-line therapy, but did recommend as a potential consideration after other pharmacological interventions failed to achieve clinical benefit. Each of the trials concluded that extended release tramadol provided clinically important pain relief in chronic LBP, over 4- and 8- weeks, however, further research is needed to determine long-term safety and efficacy [87].

At least four systematic reviews have been conducted with interest in duloxetine for chronic LBP [89–92] with consensus that that duloxetine had modest to moderate effects on pain relief, functional improvement, mood regulation, and/or improvement in quality of life. Although not cited in any CPGs we included, a Cochrane review of antidepressants for non-specific LBP [93] evaluated seven studies which compared TCA and SSRI antidepressants with placebo and were considered low risk of bias [83, 94–99]. Of the seven studies, five reported no difference in pain between antidepressants and placebo [83, 94, 95, 97, 98]. A more recent meta-analysis of antidepressants for LBP included 16 trials and determined the evidence ranged from low to very low for pain and disability across follow-up periods and any benefit achieved at 2 weeks was not clinically meaningful [100].

In a recent systematic review of medications for LBP, acetaminophen, antibiotics, SMRs, TCA, duloxetine, anticonvulsants, NSAIDs, and/or opioids, only baclofen (a SMR), duloxetine, NSAIDs, and opioids (including tramadol) had evidence of improved pain and disability in patients with chronic LBP [89]. The review recommended future research is needed to determine long-term efficacy and safety. Consistent with the CPGs from the last 5 years, additional research is needed, in particular, when considering chronic LBP. Expert opinion is heavily relied upon due to a lack of high-quality prospective trials evaluating long-term use for these medications.

Finally, SMRs for acute LBP lacked consistency of recommendation across CPGs and even with a more recent systematic reviews [101, 102]. Abdel Shaheed et al. highlight that the disagreement in recommendation maybe stems from evidence of statistically significant pain relief compared with placebo which may not translate to clinically important effects [101], while Cashin et al. noted increased risk of adverse event and determined very low to low certainty evidence shows that SMRs fall short of clinically important reductions in pain intensity for acute LBP [102]. In taking into consideration the potential for abuse and sedative effects of SMRs, it was concluded there were insufficient outcomes to support utilizing SMRs for acute LBP [101].

### Strengths and weaknesses

For this study, only CPGs in the English language were evaluated, which may have excluded non-English CPGs offering additional insight. Despite this restriction, there was global representation of non-primary English-speaking countries with CPGs from Belgium [72] and Denmark [74], and the Global Spine Care Initiative included working group members from Chile and Switzerland [62]. Jurisdictional differences in payment structures and treatment availability may have contributed to the variability between CPGs. A greater importance may have been placed on cost and accessibility over efficacy depending on the intended population for use (e.g. The Global Spine Care Initiative: applying evidence-based guidelines on the non-invasive management of back and neck pain to low- and middle-income communities) or the necessity of cost-effectiveness and benefits research of considered interventions when deciding on recommendations (e.g. NICE) [103].

With only evaluating CPGs, we therefore excluded all systematic reviews, which may have provided additional insight into the effectiveness of individual medication classes. Furthermore, the included CPGs may have relied on other synthesized reviews to inform their recommendations instead of RCTs. This may have contributed to the recommendation of medication classes that lack supporting evidence of effectiveness, but have been historically recommended due to low potential for harm (e.g. acetaminophen).

Changes in clinical practice due to the opioid epidemic has resulted in increased clinical use of duloxetine for concomitant LBP and depression or anxiety [62].Concerningly, all the RTCs cited by the CPGs for the efficacy of antidepressants and chronic LBP were performed between 1976 and 2007 and many of these did not include duloxetine.

Finally, there will always be difficulty with studying a condition such as non-specific LBP due to patient and condition heterogeneity, natural history, a supermarket of approaches to management, and/or varying diagnostic definitions between researchers or providers.

### Future directions

The CPGs reviewed in this paper demonstrate the wide variability of recommendations for both acute and chronic non-specific LBP. In the wake of the opioid epidemic and the persistence of LBP as a global disability burden, identifying the most effective and safe medications is paramount. Future research should seek to establish consensus on guideline-recommended care for both acute and chronic LBP. It is evident that further investigation is needed to determine both the efficacy and long-term safety of duloxetine, tramadol, and skeletal muscle relaxants. Benzodiazepines and antibiotics recommendations from the evaluated CPGs indicate there is limited value in spending additional resources to further evaluate these pharmacological strategies for LBP due to the large side effect profile coupled with limited clinical benefit. Although primary research is limited, cannabis and cannabinoid derivatives are commonly seen in clinical practice, particularly as an alternative analgesic to opioids, and could be considered for future CPGs [104].

While recommending medications that have little-to-no demonstrated clinical benefit (e.g. acetaminophen) may be a consideration due to the accessibility, low cost, and minimal potential for harm, we believe that it is important to highlight the need for full transparency regarding its anticipated efficacy. When recommending acetaminophen to a patient, it should be paired with education, encouragement for movement, and recommended non-pharmacological care interventions and not reliance on the medication itself to produce clinically significant benefit for the patient.

## Conclusions

To manage acute and chronic LBP, NSAIDs are recommended as a first-line medication, whereas, no CPGs recommend antibiotics, anticonvulsants, benzodiazepines, or oral corticosteroids. There is little agreement amongst CPGs in recommendations for antidepressants, acetaminophen, opioids including tramadol, and SMRs. Disagreement is likely a reflection of the varying weight placed by the individual guideline experts in interpreting the supporting literature, accessibility/cost, and consideration of potential harms.

## Supplementary Information

**Additional file 1: **Evidence tables for acute low back pain.

**Additional file 2: **Evidence tables for chronic low back pain.

## Data Availability

The datasets used and/or analyzed during the current study are available from the corresponding author on reasonable request.
